# Negative correlations between anxiety-depressive symptoms and quality of life among patients on hemodialysis

**DOI:** 10.1590/S1516-31802010000200012

**Published:** 2010-03-04

**Authors:** Juliana Bonfim do Santos, Melina Mendonça, Maria Carolina Pedalino Pinheiro, Sergio Tamai, Ricardo Uchida, Luiz Antonio Miorin, Marsal Sanches

**Affiliations:** I Medical student, Faculdade de Ciências Médicas da Santa Casa de São Paulo (FMSCSP), São Paulo, Brazil.; II MD. Psychiatric resident, Integrated Mental Health Center, Santa Casa de São Paulo, São Paulo, Brazil.; III MD, PhD. Professor in the Department of Psychiatry, Faculdade de Ciências Médicas da Santa Casa de São Paulo (FMSCSP), São Paulo, Brazil.; IV MD, PhD. Professor in the Department of Internal Medicine, Faculdade de Ciências Médicas da Santa Casa de São Paulo (FMSCSP), São Paulo, Brazil.

Dear Editor,

Psychiatric symptoms such as anxiety, depressive symptoms and cognitive impairment are extremely common among patients with chronic renal failure and several studies have focused on the impact of these symptoms on the quality of life of patients on hemodialysis.^1-5^

In order to address this issue, we conducted a cross-sectional study in the Renal Unit of Santa Casa de São Paulo Central Hospital. The sample consisted of 23 patients with chronic renal failure on maintenance hemodialysis (10 male and 13 female; mean age = 39.3 ± 12.8 years; mean duration of dialysis treatment = 68.9 months). The patients were administered the Beck Depression Inventory (BDI), Beck Anxiety Inventory (BAI), Mini-Mental Status Examination (MMSE) and World Health Organization Quality of Life Instrument (WHOQOL). The study was approved by the respective Institutional Review Board and written informed consent was obtained from all patients prior to their inclusion in the study. The data were analyzed using the Statistical Package for the Social Sciences (SPSS) version 10.0. We calculated the Spearman correlation coefficient between the scores on the anxiety and depression scales and the WHOQOL scores.

The statistical analysis revealed a moderate negative correlation between the BAI and WHOQOL scores (r = −0.39; P < 0.01), as well as a moderate negative correlation between the BDI and WHOQOL scores (r = −0.54; P < 0.01). On the other hand, no statistically significant correlation was found between the MMSE and WHOQOL scores.

Our findings seem to imply a negative correlation between anxiety and depressive symptoms and the quality of life among patients on hemodialysis. These findings are in consonance with the results of previous studies.^1-3^

With regard to methodological limitations, the negative results relating to quality of life and MMSE performance may be due to the small sample size. In addition, we cannot exclude the role of confounding factors, since patients who scored lower in the MMSE might have found difficulties answering the WHOQOL. Nevertheless, regardless of these methodological issues, our findings highlight the importance of adequate identification and management of psychiatric symptoms among patients with chronic renal failure.
